# Green photosensitisers for the degradation of selected pesticides of high risk in most susceptible food: A safer approach

**DOI:** 10.1371/journal.pone.0258864

**Published:** 2021-10-28

**Authors:** Ayesha Baig, Muhammad Zubair, Sajjad Hussain Sumrra, Umer Rashid, Muhammad Nadeem Zafar, Fayyaz Ahmad, Muhammad Faizan Nazar, Mujahid Farid, Muhammad Bilal, Fahad A. Alharthi, Dimitrios A. Giannakoudakis

**Affiliations:** 1 Department of Chemistry, University of Gujrat, Gujrat, Pakistan; 2 Institute of Advanced Technology, Universiti Putra Malaysia, UPM, Serdang, Selangor, Malaysia; 3 Department of Statistics, University of Gujrat, Gujrat, Pakistan; 4 Department of Chemistry, University of Education Lahore, Multan Campus, Multan Pakistan; 5 Department of Environmental Sciences, University of Gujrat, Gujrat, Pakistan; 6 School of Life Sciences and Food Engineering, Huaiyin Institute of Technology, Huaian, China; 7 Chemistry Department, College of Science, King Saud University, Riyadh, Saudi Arabia; 8 Institute of Physical Chemistry, Polish Academy of Sciences, Kasprzaka, Warsaw, Poland; 9 Department of Chemistry, Aristotle University of Thessaloniki, University Campus, Thessaloniki, Greece; Bangabandhu Sheikh Mujibur Rahman Agricultural University, BANGLADESH

## Abstract

Pesticides are the leading defence against pests, but their unsafe use reciprocates the pesticide residues in highly susceptible food and is becoming a serious risk for human health. In this study, mint extract and riboflavin were tested as photosensitisers in combination with light irradiation of different frequencies, employed for various time intervals to improve the photo-degradation of deltamethrin (DM) and lambda cyhalothrin (λ-CHT) in cauliflower. Different source of light was studied, either in ultraviolet range (UV-C, 254 nm or UV-A, 320–380 nm) or sunlight simulator (> 380–800 nm). The degradation of the pesticides varied depending on the type of photosensitiser and light source. Photo-degradation of the DM and λ-CHT was enhanced by applying the mint extracts and riboflavin and a more significant degradation was achieved with UV-C than with either UV-A or sunlight, reaching a maximum decrement of the concentration by 67–76%. The light treatments did not significantly affect the in-vitro antioxidant activity of the natural antioxidants in cauliflower. A calculated dietary risk assessment revealed that obvious dietary health hazards of DM and λ-CHT pesticides when sprayed on cauliflower for pest control. The use of green chemical photosensitisers (mint extract and riboflavin) in combination with UV light irradiation represents a novel, sustainable, and safe approach to pesticide reduction in produce.

## Introduction

Vegetables are the edible and fresh part of herbaceous plants important for the diet, providing a plethora of minerals and vitamins for humans [1, 2]. Cauliflower (*Brassica oleracea*) is a highly consumed vegetable, enriched with nutraceuticals that help to prevent physical damage against oxidation [3]. Cauliflower is rich in dietary fibers, minerals, proteins, vitamins, and phenolic compounds with antioxidant properties [4]. Cauliflower is one of the major vegetables cultivated in the subcontinents and is mainly produced and consumed in winter, and it is also the most common vegetable in Pakistan because of a better economic return on investment. The Sindh province in Pakistan is famous for cauliflower production. Horticultural crops contribute to about 22% of the national food production and 6% of the country’s GDP, with the production reaching 5675.8 thousand tonnes annually [5].

Pesticides are used globally to protect food and fibre, and the rational recommendations of pesticides to require that they not only provide effective pest control but, at the same time, their residues in commodities or agricultural products should be toxicologically acceptable. Food safety is of paramount importance to the global food supply chain [6, 7]. Pesticides are used for repelling, destroying, or preventing pests but if their residues remain in the crops, these pesticides result in serious health hazards, such as causing immune dysfunction, Parkinson’s disease, cancer, toxicity in children, and cytotoxic defects [8, 9]. The use of different pesticides on crops, without respecting recommended time intervals and good agricultural practices result in pesticide residue accumulation in the inner parts of crops or vegetables. Due to the consumption of vegetables in semi-processed or raw form, they may hold a comparatively higher amount of pesticide residues, including breads and cereals. The accumulation of pesticide residues in humans can cause health hazards such as haemangioma, nervous system problems, cardiovascular disorder, orofacial clefts, gastrointestinal or musculoskeletal pathologies. Thus, with the consideration of the hazardous effects of pesticides through the consumption of cauliflower, the quantification of pesticide residues and their health implications were explored by collecting cauliflower samples from peri-urban areas of Faisalabad, Pakistan [10].

Pesticides, including λ-CHT, have been used to analyze the bio efficacy against Diamondback moths which were found to be relatively good [11]. There are different routes regarding the accumulation of pesticides with the most common to be via the direct intake or consumption of the fresh foods. Therefore, to limit pesticide residues from fresh foods or agricultural products towards the improvement of food items’ safety, various methods or techniques have been developed and studied in detail in recent decades. Many chemical-based cleaning methods, such as chlorine treatment and the use of surfactants, ionic solvents, and ozone, have been used to decrease the pesticide residues’ concentration. However, none of these commonly used techniques has been described as a successful one to eliminate pesticide residues without chemical and/or physical side-effects on the food itself. Therefore, there is a fundamental necessity to explore more sustainable, environment-friendly, and effective pesticide removal methods [12].

Lambda-cyhalothrin is in the group of pyrethroid insecticides, which are synthetic chemicals that are chemically similar to the natural insecticides known as pyrethins. These insecticides are widely used for controlling pests in gardens, homes, public health areas, and in general agriculture in Pakistan [13]. Ideally, any residual insecticides, such as DM and λ-CHT, should be bio-degraded after successfully killing the pests in cauliflower, since they may affect the antioxidants of the cauliflower. These antioxidants, naturally present in cauliflower, are beneficial because they exhibit anti-bacterial and anti-inflammatory actions in the human body [14]. Regional or national authorities conduct the dietary risk assessments for the pesticides, during the process of registration for the establishment of the legal or permissible maximum residue limits for the agricultural commodities [15]. For consumers’ protection from the intake of unsafe levels of pesticides, the Food and Agriculture Organization of the United Nations and the World Health Organisation have established MRLs (Maximum Residue Limit) for pesticides in a variety of foods, as set out in the Codex Alimentarius. For ensuring food safety and food quality, a simple and rapid analytical method with high accuracy in quantitation and identification of pyrethroid residues is necessary to regulate and monitor the usage of such pesticides [16].

The degradation of deltamethrin in both *in vivo* and *in vitro* models by UV light irradiation is an efficient method for degrading the residues of the pesticides in cauliflower. But this may result in a change in fatty acid, phenolic acid/organic acid profile, flavonoids, and other nutritious contents naturally present in cauliflower [17]. Similarly, photo-degradation is also an effective tool for the degradation process of λ-CHT along with the determination of its degradation rates [18]. In this research, the photodegradation of the DM and λ-CHT residues in cauliflower was assessed in combination with green chemical photosensitisers, mint extract and riboflavin, and a risk assessment and the effect on natural antioxidants were also evaluated.

## Materials and methods

### Procurement of the pesticides, chemicals, and samples

In the present research work, the DM and λ-CHT pesticides were obtained from the Tara Group (Lahore, Pakistan). DPPH (2,2-diphenyl-1-picrylhydrazyl), BHT (butylated hydroxy toluene), acetonitrile, Folin-Ciocalteau reagent, gallic acid, sodium carbonate, sodium hydroxide, hydrochloric acid, riboflavin, and methanol were used of analytical grade purity level and were procured from Sigma-Aldrich (Germany). Samples of the pesticide free cauliflower (n = 5, each 1.0 kg) were randomly collected from a controlled experimental field irrigated with freshwater at Gujrat, Pakistan. The procured samples were kept in airtight polyethylene bags and brought to the research laboratory Department of Chemistry, University of Gujrat for further analysis.

### Study design

Treatments included untreated cauliflower, cauliflower samples sprayed with 20, 40, 60, 80, or 100 mg/L of each pesticide, with or without either mint extract or riboflavin (1 mg/L) and these same samples exposed to one of three light sources for 180 min with subsample removed every 10 min. Samples were extracted and the total phenolics, antioxidant activit, and residual pesticide content were measured.

### Pesticide treatment and light exposure conditions

Fresh cauliflower samples were washed with distilled water to clean them from dust and dirt. Moreover, the water was removed by surface air drying on filter paper. Then the whole cauliflower curd (edible portion) was sliced into small pieces of about 1–2 cm in length, with a sharp stainless-steel knife for further processing, whilst the non-edible parts, consisting of stalks, leaf midribs and stem, were discarded [1]. The edible portion of the cauliflower was then divided into three equal portions. The cauliflower samples were sprayed with 20, 40, 60, 80, or 100 mg/L of each pesticide. with the addition of 1 mg/L of mint and riboflavin, separately. The samples of the treated cauliflower were exposed to the light sources and subsamples were withdrawn after 20 min, 40 min, 60 min, 120 min and 180 min.

In a separate experiment, 100 mg/L concentration of each pesticide, with and without the addition of 1 mL of 100 mg/L riboflavin or mint solution, was exposed to UV-A, UV-C, and sunlight and the samples were collected after 180 min.

### Methanol extraction

The dry powder form of the cauliflower was prepared by cutting of the cauliflower into further small pieces which were then dehydrated by hot air oven drying [19] at 50°C for 24 hrs. The dehydrated cauliflower was then ground into a fine powder with the help of a mortar and pestle. 10 g of powdered cauliflower was mixed with 50 mL of methanol in an orbital shaker for 24 hrs. Then the extract was filtered over filter paper (Whatman no. 1) and the filtrate was collected. The extract was placed into the sample vials, and stored at 5°C by way of the preservation protocols of Mandal & Singh [20] with some modifications, for further analysis.

### Antioxidant activity

The total phenolic contents of the cauliflower (both untreated and treated samples) extracts were estimated by using the Folin-Ciocalteau reagent [21, 22]. Different concentrations (0.1, 0.2, 0.4, 0.6, and 0.8) of both the untreated and treated cauliflower extracts (in methanol) were taken separately. The absorbance of all the samples was measured, in a triplicate manner, at 760 nm by using a UV-Vis Spectrophotometer (Lambda EZ 201, Perkin Elmer, USA), against a reference standard of gallic acid (with varying concentrations of 100, 200, 400, 600, 800, and 1000 mg/L). The results were calculated in the form of gallic acid equivalent (GAE, mg/g). The DPPH radical scavenging activity of the untreated and treated cauliflower extracts was assessed by using the reported method [23, 24], with some modifications. The results were obtained in the form of percent inhibition, compared with the BHT standard with a range of concentration from 100–1000 mg/mL.

### Analysis of the pesticides by using HPLC and GC-MS

The purity of the pesticide standards was verified by GC-MS (QP-2010, Shimadzu, Japan) to ensure the quality of the work (S1 and S2 Figs). The concentrations of the pesticides in the control and treated samples (before and after light) were analysed by using reverse phase HPLC (2010 Prominence Shimadzu, Japan). In this protocol: 20, 40, 60, 80, and 100 mg/L solutions of DM (in acetonitrile) were exposed, with and without the addition of the photosensitisers (mint and riboflavin), to the UV-A, UV-C, and sunlight for degradation. Then samples were taken out after different time intervals of 20, 40, 60, 120, and 180 min, in sample vials and were analysed by using RP-HPLC with UV detection, which was proved as a good alternative method for the determination of the pesticides. The chromatographic separations with C_18_ columns produced significant results, and a UV-visible detector set at 267 and 254 nm for DM and λ-CHT, respectively, offered efficient and suitable chromatograms for quantification or analysis of DM and λ-CHT in real samples. The peak areas of the pesticide standards were recorded and the concentrations of the DM and λ-CHT were determined with respect to their peak areas.

### Risk assessment

A dietary risk assessment of the DM and λ-CHT in the cauliflower samples was based on the data obtained from the degradation of the pesticides using UV light and sunlight. The nutritional values of the cauliflower extracts were compared and analysed before and after light application. A high concentration (100 mg/mL) was used for detection purposes. The risk assessment was also estimated in the form of a hazard index (HI) which was calculated by dividing the estimated daily intake (EDI) and ADI. After the light application, the HI values were calculated for the DM and λ-CHT. These values were than compared with the safe levels of pesticide residues present in cauliflower [25].


$$HI=\frac{EDI}{ADI}$$

### Statistical analysis

Statistical analyses (ANOVA 2 factor without replication test and MANOVA) were used to check the significant differences between the factors (time and concentration) on the degradation of the pesticides and the change in the TPCs and antioxidant properties.

## Results and discussion

The present research study is related to the sustainable photo-degradation of DM and λ-CHT using green photosensitisers and the effect on the antioxidant attributes of cauliflower.

### Effect of light and green photosensitisers on the extraction yield of cauliflower

The extraction yield of the cauliflower (untreated group) was 14.0 g/100 g. The extraction yields of the cauliflower samples treated with DM, before and after UV exposure, were 17.33 g/100 g and 15.01 g/100 g, respectively, which are in agreement with those reported by Prodhan, et al. [26]. Similarly, for the cauliflower samples treated with λ-CHT, the extraction yields, before and after exposure to the UV light, were 18.05 g/100 g and 16.51 g/100 g, respectively. The present results (Table 1) show a considerable variation of extraction yields of the cauliflower treated with the pesticides (DM and λ-CHT) before and after exposure to light. A higher yield was achieved after spraying the pesticides on the cauliflower, while light (sunlight or UV) exposure led to lower yield values. The increase in the yield of extraction after treatment supports the extraction of the pesticides along natural extracts. The decrease in extraction yields up to 13% of the cauliflower after exposing the samples to light clearly shows that light led to the degradation of pesticides, whereas the method of extraction was the same for all the samples. These results were supported by some other studies of pesticide degradation which may be due to applying the UV-irradiation [27, 28]. These variations in the extraction yields are in comparison with those reported by Anwar, et al. for the extraction of cauliflower in 100% methanol as the extraction solvent [29]. In the literature, research studies hardly offered evaluation of the extraction yields as has been presented in this present study.

**Table 1:** Extraction yield (%) in methanol of cauliflower with and without pesticides and before and after light effect.

**Treatment**	**Yield %**	
Untreated	14.00 ± 0.60	
	**DM Treated**	**λ-CHT Treated**
No Light	17.33 ± 0.78	18.05 ± 0.92
Sunlight	16.01 ± 0.90	17.51 ± 0.73
UV-A	15.01 ± 0.82	17.11 ± 0.68
UV-C	14.01 ± 0.68	16.51 ± 0.67

### Effect of light and photosensitisers on the cauliflower’s antioxidants

#### Effect of total phenolic contents

**Fig 1:**
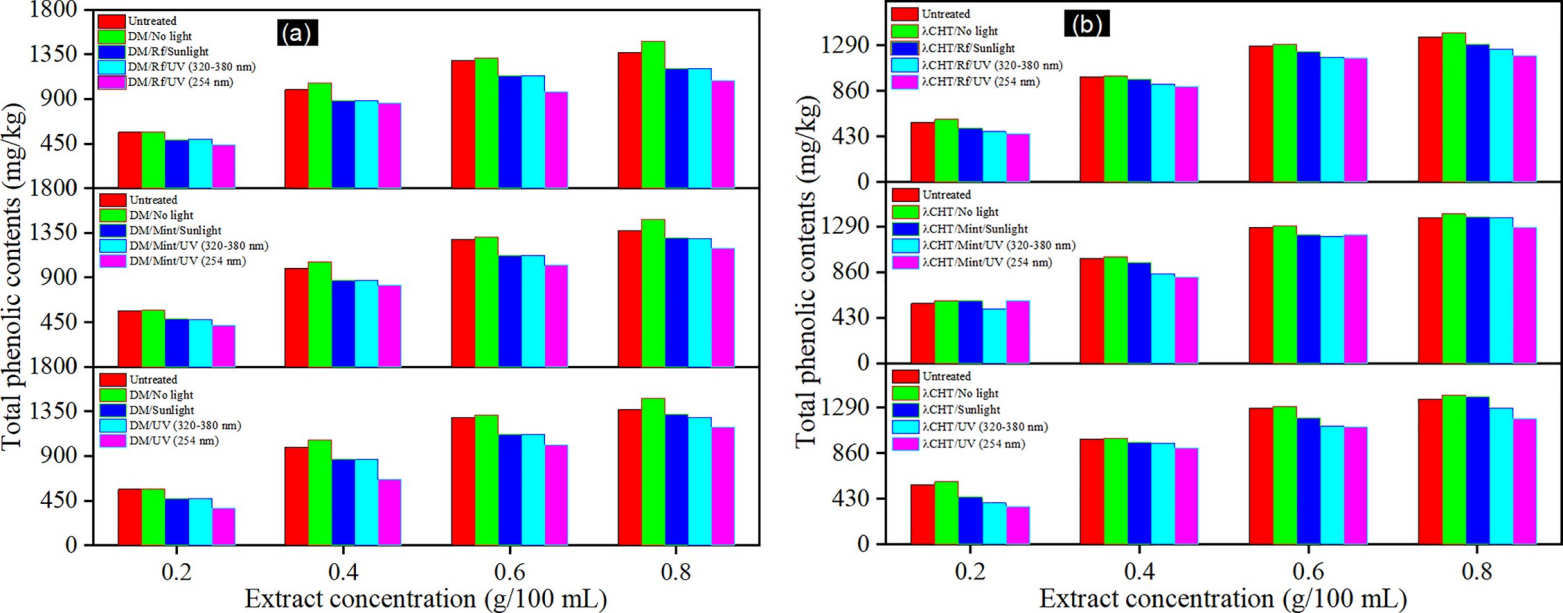
Effect of light and green photosensitizers on TPCs of cauliflower with and without pesticides i.e., (a) DM, (b) λ-CHT.

Several researchers have found a significant correlations between total phenolic contents (TPCs) and antioxidant activity, by employing the DPPH radical scavenging assay in samples of cauliflower [30]. It has been confirmed that cauliflower has a high enough level of antioxidants. Hence in the present study, the results of the total phenolic contents (TPCs) are depicted in Fig 1(A) and 1(B). The effect of pesticides, light, and green photosensitisers brought to light noteworthy results of the TPCs of the cauliflower extracts. Using 0.2 to 0.8 g concentrations in methanol, the total phenolic contents of the untreated and the pesticide (DM and λ-CHT) treated samples, before and after light and catalytic application, were determined. Overall, not a very significant effect on the TPCs was found in respect to the degradation under light exposure. The results also show that spraying the DM pesticides on the cauliflower enhanced the TPCs of the extracts by up to 8% which may be the reason of salting out due to pesticides. A set of cauliflower extracts after pesticide application was also treated with different types of lights and a maximum 16% decrease in the TPCs was found under sunlight, 26% under UV-A, and 34% under UV-C. Similarly, the application of green photosensitisers (mint extract and riboflavin) along with different lights also effected the TPCs of the cauliflower but the effect was not enough to be considered significant in respect to the decrease in the TPCs. In the presence of mint as the photosensitizer, different light sources had an effect on the TPCs at 15–26% whereas this effect was 14–25% with the riboflavin mediation. Secondly, the effect of light on the TPCs with the λ-CHT application was also determined and a similar trend was observed as in the DM. An amount of 4% TPCs of the cauliflower extract was increased when the λ-CHT was applied which was then decreased by using the different lights in the presence of the photosensitisers. A decrease from 22 and up to 38% was observed in the use of different lights whereas 19 ± 1% was observed in the presence of the photosensitisers, i.e., mint and riboflavin. Overall, it was observed that only the light irradiation effected more on the TPCs of the cauliflower whereas the green photosensitisers sustained the TPCs decrease either by self-activity or by absorbing the light. Initially, there was an increase in the TPCs after spraying the formation of the various types of volatile organic compounds and the salting out effect with the pesticide, whilst, after the application of the light, these values were decreased with the degradation of the pesticides [27, 31]. The increase of the TPCs was dependent on the special properties of the insecticides [32]. These findings are in agreement with those reported by Bragança et al. [33] and Abd-ur-Rahman, et al. [34], according to which, the most effective insecticides against aphids and whiteflies and for increasing TPCs were cyhalothrin followed by Imidacloprid. Bt spraying with DM and λ-CHT on the cauliflower, there was no significant difference (p value > 0.05) in the TPCs or on the antioxidant attributes of the treated and untreated samples of the cauliflower, and with all the three light sources (sunlight, UV-A, and UV-C) used [35].

#### Effect on radical scavenging activity

The cauliflower’s DPPH radical scavenging activity was expressed in percent inhibition of the DPPH radical by the cauliflower extracts. A similar scheme of work was used to express the effect of the light and photosensitisers on the in-vitro antioxidant potential of the cauliflower for both the samples untreated and treated with the pesticides. In the untreated cauliflower, the percent inhibition ranged from 40.74% to 94.27% in a range of 0.2–0.8 g concentrations of the extracts. Whilst in the case of spraying the DM and λ-CHT on the cauliflower samples, the percentage inhibition ranged from 40.32–90.48% and 45.37–91.51%, respectively. After exposing the treated samples to sunlight, UV-A and UV-C light for 20–180 min, the percent inhibition was observed in the ranges from 30–76% for DM and 33–78% for λ-CHT, respectively (Table 2). A non-significant effect in the decrease of the scavenging activity of the cauliflower was observed. Light had the maximum effect up to a 35% decrease in the radical scavenging activity. UV-C light having a wavelength of 254 nm with more energy radiation imparted the major effect, but this effect had been decreased due to the green photosensitisers (mint and riboflavin). It is fascinating to apply the green photosensitisers for pesticide degradation with a sustainable approach. These results are in close agreement and more pronounced than the 68.91%, 58.93%, 59.15%, and 61.83% for the stir-fried, steam-boiled, steam-blanched, and fresh cauliflower extracts, respectively, reported by Ahmed & Ali, to investigate the effect of a number of different cooking methods on the antioxidant activities of cauliflower [36]. With increasing the degree of the hydroxylation or the concentration of the polyphenols, the DPPH radical scavenging activity also increased. Anwar et al. [29] obtained cauliflower extracts from oven-dried, air-dried, and sun-dried samples and concluded that they exhibited an appreciable percent inhibition in the range of 62.6–70%. Inother study, Koksal and Gulcin [14], reported the scavenging activities of about 64 and 51% for ethanol and water cauliflower extracts, respectively. These percent inhibition values are in agreement with present study results.

**Table 2:** Effect of light and green chemicals on the percent DPPH radical scavaging activity (range %inhibition) of cauliflower extracts (concentration of extract here) with and without pesticides application.

Photosensitisers Concentration	Untreated	Pesticide Treated	Light Source	No photosensitizers	Mint	Riboflavin
0.2–0.8	46.12–94.28	**DM**	Sunlight	35.32–69.84	43.32–76.41	40.32–64.73
		40.32–90.48	UV-A	34.92–68.22	42.49–74.64	41.82–65.98
			UV-C	30.03–60.18	36.17–70.97	30.12–64.55
		**λ-CHT**	Sunlight	33.87–70.01	42.37–78.51	35.37–71.44
		45.37–91.51	UV-A	33.87–66.45	43.19–71.66	34.45–69.55
			UV-C	34.35–70.50	41.65-67-21	32.46–65.86

#### Effect of the light and green photosensitisers on pesticide degradation

**Fig 2:**
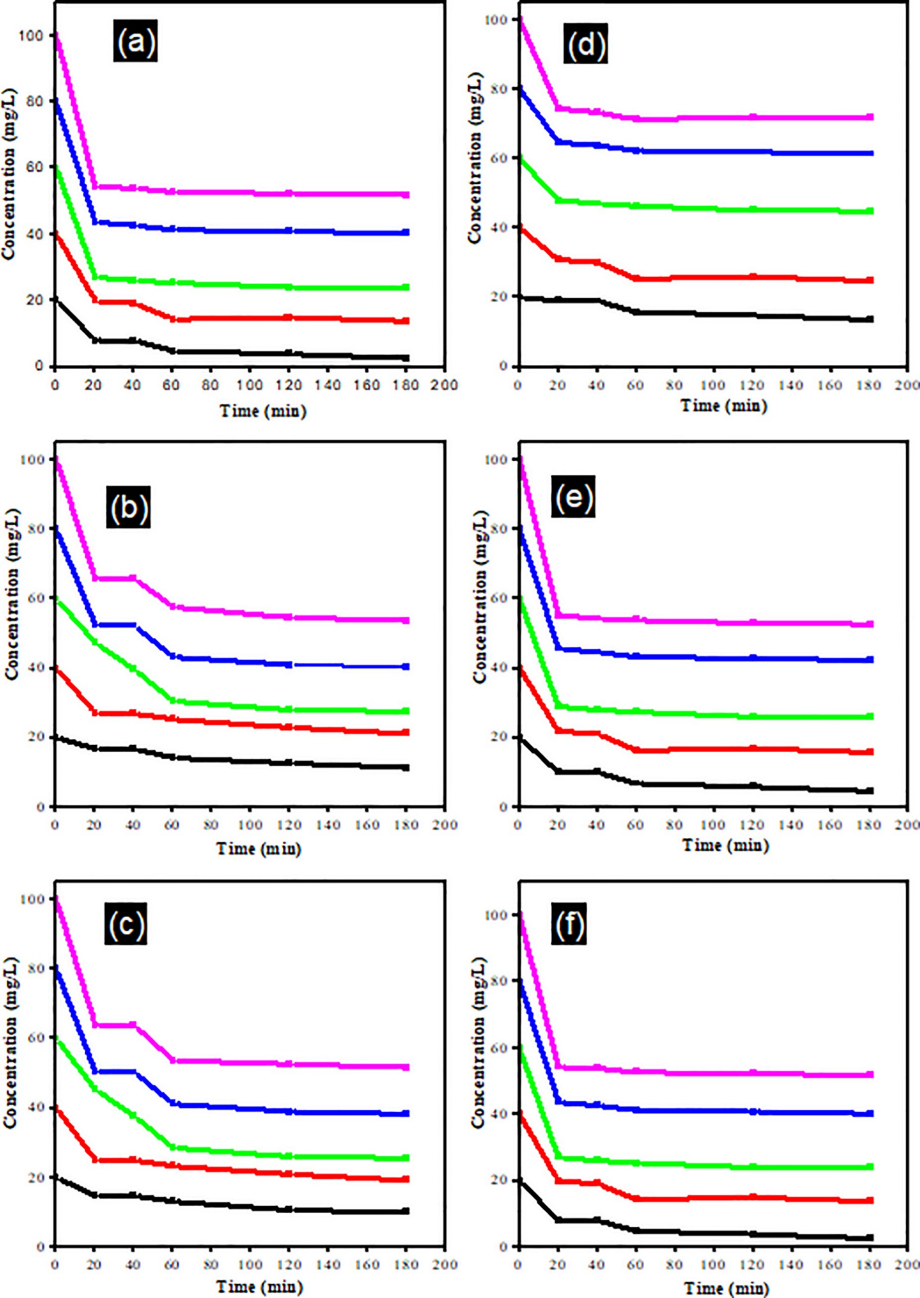
Effect of light on DM and λ-CHT pesticides degradation a) DM + Sunlight; b) DM + UV-A; c) DM + UV-C; d) λ -CHT + Sunlight; e) λ -CHT + UV-A; f) λ -CHT + UV-C.

**Fig 3:**
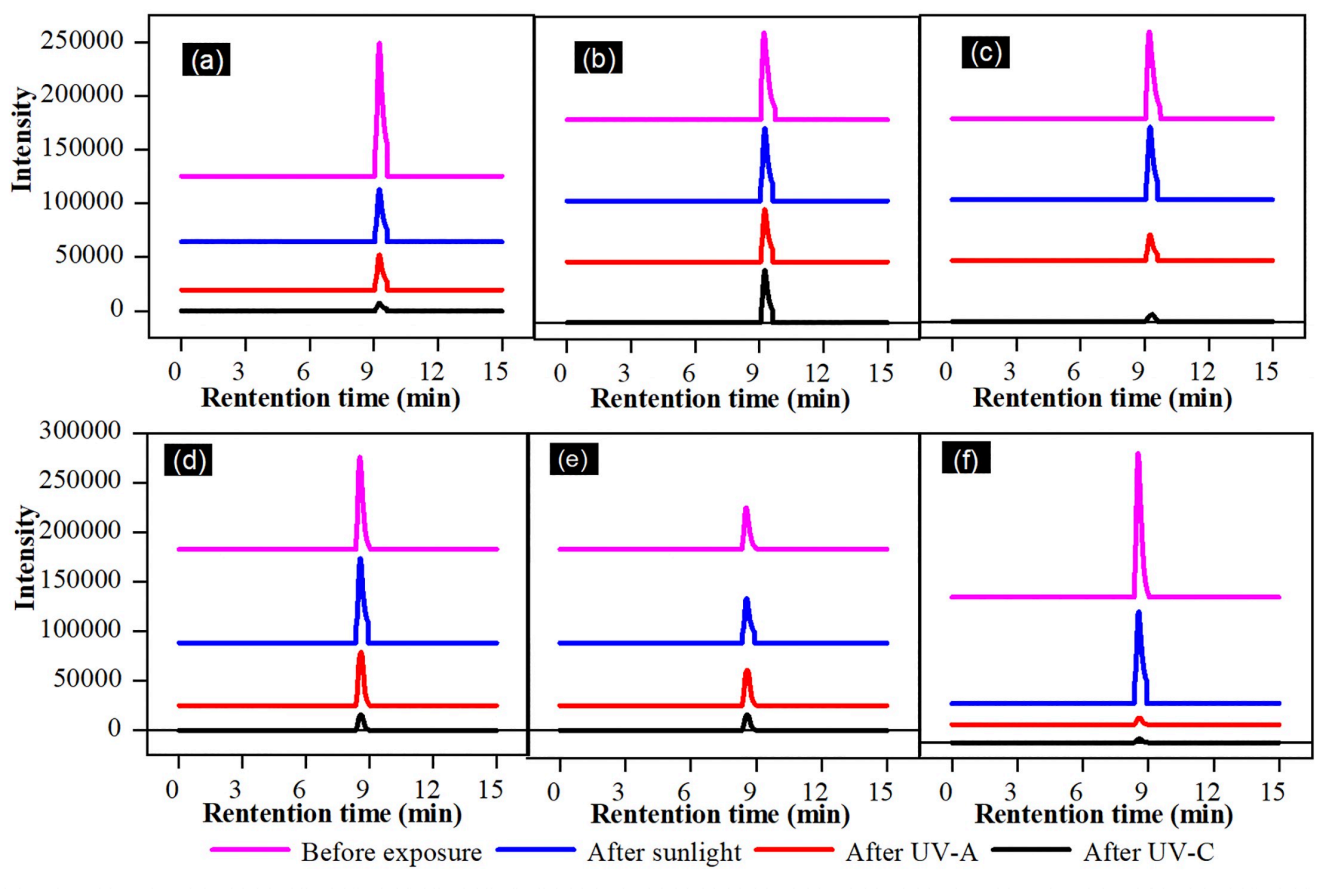
Effect of light and photosensitizers on DM and λ-CHT pesticides degradation a) DM + Light; b) DM + Light + Mint; c) DM + Light + Riboflavin; d) λ-CHT + Light; e) λ-CHT + Light + Mint; f) λ-CHT + Light + Riboflavin.

Fig 2 shows the photo-degradation of the λ-CHT (20 to 100 mg/L) at different time intervals and different light irradiation mediations with the green photosensitisers. No significant change after 180 min of light was observed in the DM concentration in the solutions without the photosensitisers, i.e., mint and riboflavin, indicating that the direct photo-degradation process initiated by a photosensitiser could be considered on a time scale [37]. In the case of the 100 mg/L solution of DM, the best degradation results (32.0 mg/L) were obtained by using mint as the photosensitiser and on exposure to the UV-C light. Whilst the highest degradation of the λ-CHT (24.2 mg/L) was obtained by using riboflavin as the photosensitiser for the degradation and under the UV-C the (Fig 3). Without the addition of natural, low-cost, and nontoxic photosensitisers, the degradation process was comparatively slower with less degradation of the DM (65.92 mg/L) and λ-CHT (56.6 mg/L) being observed. Comparatively, the best degradation results were obtained in the case of using UV-C as a light source to degrade the DM and λ-CHT. In comparison to both pesticides being used, the highest degradation was obtained by the λ-CHT (24.2 mg/L) which was in comparison with those reported by Akomea-Frempong, et al. [25], Reddy & Reddy [38], and Ramarao & Goud [39], respectively. The use of natural photo-initiators of the degradation process, such as mint and riboflavin, makes the whole degradation process a safe method to reduce pesticide residues in cauliflower, and they also enhance the nutritional contents of the cauliflower. In a similar research study, the degradation of λ-CHT, without the addition of photo-initiators (Cu^2+^ and humic acid), was observed to a certain time extent. The photo-degradation of the λ-CHT was rapid within the first 1 hr and then this process became slower and the removal efficiency of 59.3% was comparable to this study [37]. In another research study, UV/Vis and GC-MS were used to evaluate the degradation of of λ-CHT in laboratory-controlled soils and it was reported that the degradation was initially fast in the first few days through photolysis and other chemical processes. The subsequent dissipation rate showed a steady slow decline, which could be evidence of microbial degradation [40].

Riboflavin is non-toxic, naturally occurring in the human body, and a sole initiator of light in the range of 220–450 nm. Riboflavin is a water-soluble vitamin with photosensitising and redox properties. It is an attractive alternative to many synthetic photo-initiators. The photochemical properties of riboflavin are due to a system of conjugated double bonds and a 7,8-dimethyl-10-(1′-D-ribityl) isoalloxazine fragment present in it [41]. In the presence of oxygen and light, riboflavin generates O_2_^-^ radicals, which then subsequently initiate the degradation of pesticides [42]. Riboflavin undergoes a range of different reactions under UV-irradiations, under chemical and thermal treatment. Under UV-irradiation, riboflavin undergoes photolysis and can produce reactive oxygen species [41].

In these photochemical reactions, riboflavin absorbs energy from the UV light and is excited to singlet state (lifetime: 10^−8^ s), which is then transformed into a triplet-excited state (lifetime: 10^−2^ s) [43]. By exposing pesticide solutions to UV light and taking samples after different time intervals for the HPLC analysis, it was concluded that the DM and λ-CHT, without adding any photo-initiator, degraded up to 40 min of the UV-exposure, whilst after this time duration, there was a need for the natural photo-initiator to degrade it up to 1.5 hrs. In comparison, the addition of the photo-initiators like mint and riboflavin in the DM solutions caused more degradation, i.e., 65.92 mg/L degraded up to 32.06 mg/L and 38.41 mg/L, respectively. Whilst in the case of λ-CHT, the addition of mint and riboflavin caused degradation of 56.62 mg/L up to 25.12 mg/L and 24.20 mg/L, respectively. Thus, the degradation efficiency of both pesticides was enhanced in the presence of mint and riboflavin but restrained in the absence of these two photo-initiators. Without the addition of the photosensitisers, the degradation efficiency was decreased. PY Liu and colleagues conducted a similar research on the degradation of the λ-CHT pesticide under UV light and sunlight. It was concluded that the addition of riboflavin as a photosensitisers sped up the process of the degradation of the λ-CHT. Thus, riboflavin is an effective photosensitiser for the degradation of λ-CHT [44]. The statistical analysis MANOVA was used to determine the significant difference between two factors (concentration and time). The significant value for factor 1 (time) and factor 2 (concentration of pesticide) was < 0.05 (0.00000253) and < 0.05 (0.000951), respectively, which means that there was a significant difference observed when increased the time duration of the UV light and the concentration of the pesticides, as illustrated in Tables 3 and 4, appropriately.

**Table 3:** Repeated measures MANOVA to identify significant variation in concentration.

Effect		Value	F	DF		P
				Num	Denom	
Concentration	Wilks’ Lambda	0.00347	23.341	12	37	0.000
	Hotelling’s Trace	82.41460	86.993	12	38	0.000
	Pillai’s Trace	1.73460	1.961	12	48	0.000
	Roy’s Largest Root	79.95700				0.000

**Table 4:** Repeated measures MANOVA to identify significant variation in time.

Effect		Value	F	DF		P
				Num	Denom	
Time	Wilks’ Lambda	0.16740	3.003	12	37	0.000
	Hotelling’s Trace	4.07405	4.300	12	38	0.000
	Pillai’s Trace	0.98705	5.483	12	48	0.000
	Roy’s Largest Root	3.84781				0.000

Another study was performed to degrade the added pesticide concentration in the soil. The degradation over time of the DM applied to cabbages was monitored and it was suggested that deltamethrin application can disturb soil and a natural biodegradation can have an important part in pesticides soil decontamination [33]. Tariq and colleagues [45] conducted the photo-degradation of the DM up to 24 hrs under UV irradiations and, as a result, about 70% of the DM concentration was reduced, which is in close agreement with the degradation pencentage (67–76%) obtained in this research study. Ahmad and Yasin [46] degraded DM up to 28%, 87%, and 97%, using a blank solution and TiO_2_ /bentonite and Cu/TiO_2_/bentonite composites, which are in comparison to the present study results (i.e., 34.08–68.00% for DM-treated cauliflower samples). The results obtained in this research study are in agreement with many other studies in the literature [17, 47]. Pandey, et al. [48] reported the maximum degradation of DM and λ-CHT of about 96.15% and 95.00%, respectively, and their half-life values were below permissible MRLs.

#### Effect of light wavelength on the pesticide degradation

The wavelength (λ) of sunlight that reaches the surface of the earth is greater than 290 nm. However, pyrethroids are unable to absorb the light with wavelengths greater than 300 nm. Since it is difficult to degrade pyrethroids directly under visible light radiation, the most widely used method is to use photosensitisers to generate hydroxyl radicals. These hydroxyl radicals then promote the degradation of the pyrethroid pesticides. The photosensitisers of the pesticides could accelerate the process of the photolysis [44]. The pesticide solutions was exposed to sunlight for 180 min and compared the results by MANOVA (Table 5). It observed that there were no significant differences when exposed the pesticide solutions to the sunlight vs the UV-A or UV-C light; the p-value obtained was > 0.05 for the sunlight and UV-A and UV-C lights. The use of the photosensitisers in the degradation of the pesticides using the UV irradiation increased the rate of the degradation [49]. Zhu and coworkers also studied the degradation pattern of the DM under UV (254 nm) irradiation and concluded that the degradation of DM is a slow process [50].

**Table 5:** Repeated measures MANOVA to identify significant variations in degradation with different light effects.

**Analysis of variance for sunlight, using adjusted SS for tests**					
**Source**	**DF**	**SS**	**MS**	**F**	**P**
Time	4	421.1	105.3	9.44	0.000
Concentration	4	13798.5	3449.6	309.44	0.000
Error	16	178.4	11.1		
Total	24	14398.0			
**Analysis of variance for UV-A, using adjusted SS for tests**					
**Source**	**DF**	**SS**	**MS**	**F**	**P**
Time	4	504.92	126.23	15.17	0.000
Concentration	4	6262.28	1565.57	188.17	0.000
Error	16	133.12	8.32		
Total	24	6900.32			
**Analysis of variance for UV-C, using adjusted SS for tests**					
**Source**	**DF**	**SS**	**MS**	**F**	**P**
Time	4	507.82	126.95	14.45	0.000
Concentration	4	6133.01	1533.25	174.57	0.000
Error	16	140.53	8.78		
Total	24	6781.35			

### Evaluation of the dietary risk assessment in the cauliflower

The persistence of pesticide residues in highly nutritious and commonly used vegetables, i.e., cauliflower, causes serious health problems when used in an untreated and uncontrolled way. Their values above an acceptable daily intake are causing many acute and chronic diseases. There are no ealier detailed studies of these pesticides including their degradation, health risks, and effects on antioxidant attributes [51]. Therefore, this research has provided a comprehensive report on the degradation of these pesticides and their residues in cauliflower and the risk assessment has been focused on the evaluation of their Acceptable Daily Intake (ADI) value. A dietary risk assessment of the DM and λ-CHT in the cauliflower samples was based on the data obtained from the degradation of the pesticides using UV light. The degradation process using the UV light decreased the level up to 67–76%. Actually, the Acceptable Daily Intake (ADI) concentration is only 0.01–0.2 mg/kg [33] in the body, which is toxic to human body. Therefore, if a healthy person of 70 kg body weight take approx. 250 g [52] of cauliflower in their daily food consumption and without degradation he should get 0.7 mg/kg daily intake of pesticide with the 0.01 mg/kg ADI (Table 6). In a similar research study, the assessment of the dietary intake (i.e., mg kg^−1^ per day) was determined as per their MPI (Maximum Permissible Intake) values, i.e., DM (0.64 mg/kg) and λ-CHT (0.064 mg/kg) [36], which are in agreement with present study results (i.e., 0.42–0.63 mg/kg and 0.10–0.15 mg/kg for DM and λ-CHT, respectively). According to Drouillet-Pinard and colleagues [53], the acceptable daily intake of λ-CHT and DM is 0.005 and 0.01 mg/kg, respectively, whilst their maximum residue limits are 0.02 and 0.05 mg/kg, respectively, and these results are in close agreement with present findings. After exposure to UV-light, the HI values obtained for the DM and λ-CHT (Table 6) were greater than 1 which indicated a safe level of pesticide residues present in the cauliflower and these findings are in close agreement with those reported by Akomea-Frempong, et al. [25]. In a similar study, it is demonstrated that the daily intake of cauliflower and many other brassica vegetables is 275 g per person in the Chinese population, which is used as the largest portion of cauliflower for the Chinese adults [54]. The residue behaviour, parent pesticide hazards, and distribution in environment and crops are well acknowledged environmental and food safety issues and have raised concerns on a large scale. Notably, many recent research studies show that there is an increasing number of pesticide residues being determined in higher frequencies and/or concentrations [55, 56]. Therefore, this research revealed that the use of radiative stress could decrease 60–70% of the pesticide residues. The exposure of diet to the residues of pesticides, exceeding maximum residue limit (MRLs) values leads to chronic and acute hazardous health effects. Therefore, steps need to be taken to ensure that the use of different pesticides during the growing stage of the food products/crops is controlled to such an extent that their residues are minimised in the food and do not risk public health [57].

**Table 6:** Risk assessment of pesticides residues in cauliflower.

Pesticide	Vegetable	^ξ^ADI (mg/kg)	ADI (mg/kg) w.r.t body weight index				^ψ^HI
			No Light	Sunlight	UV-A	UV-C	
DM	Cauliflower	0.01–0.2	0.7	0.63	0.51	0.42	˃1.0
λ-CHT	Cauliflower	0.0025–0.005	0.175	0.15	0.13	0.10	˃1.0

^ξ^ADI = Acceptable Daily Intake

^ψ^ HI = Hazard Index after Photo degradation.

## Conclusions

During the recent project, the photo-degradation of the deltamethrin (DM) and lambda cyhalothrin (λ-CHT) was studied, and the data of the pesticide residues were compared with their respective Maximum Residue Limits (MRLs). The results showed that light irradiation in addition with photosensitiser chemical mediation led to a significant degradation extent of the pesticide residues. The addition of initiators for the degradation process, like mint and riboflavin, have been also proved beneficial in making the whole degradation process as an efficient and safe way to remove or reduce the pesticide residues in cauliflower. The best degradation extent, up to 70% of both the DM and λ-CHT pesticides, was achieved with the mint extracts and riboflavin as the green photosensitisers used as degradation promoters. The degradation rate under UV-C (254 nm) was much faster than UV-A (320–380 nm) and sunlight (400–800 nm). For making the whole degradation process beneficial for human bodies, with respect to the intake of such pesticide residues present in cauliflower, natural extracts of mint leaves and riboflavin (vitamin B2) can be used as photo-degrading promoters. This safe degradation process of the pesticides does not affect the antioxidant activity of the treated cauliflower samples nor the natural nutrients of the cauliflower. So, it is highly needed to degrade their presence in crops along with the assessment of the quantity of substances that health risky for humankind. However, more detailed research on the degradation of DM and λ-CHT should be further explored.

## Supporting information

S1 Fig GC chromatogram of DM. (PDF)

S2 Fig GC chromatogram of λ-CH. (PDF)
