# Supplementary figures and images for: Green photosensitisers for the degradation of selected pesticides of high risk in most susceptible food: A safer approach
Source: PLoS One. 2021 Oct 28;16(10):e0258864. doi: 10.1371/journal.pone.0258864 (PMC8553129; doi:10.1371/journal.pone.0258864)

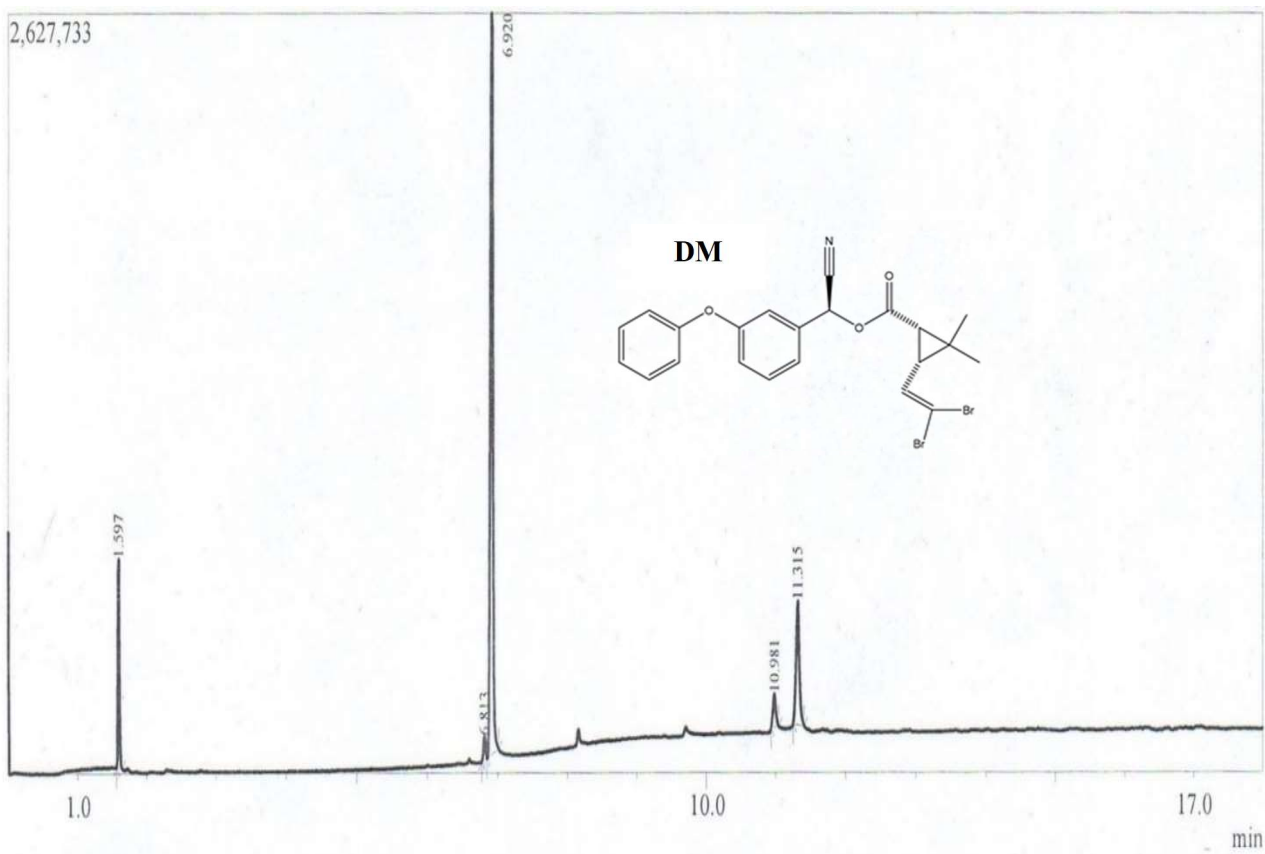

**S1 Fig. GC chromatogram of DM**

Supplement: S1 Fig — (PDF) [file pone.0258864.s001.pdf]

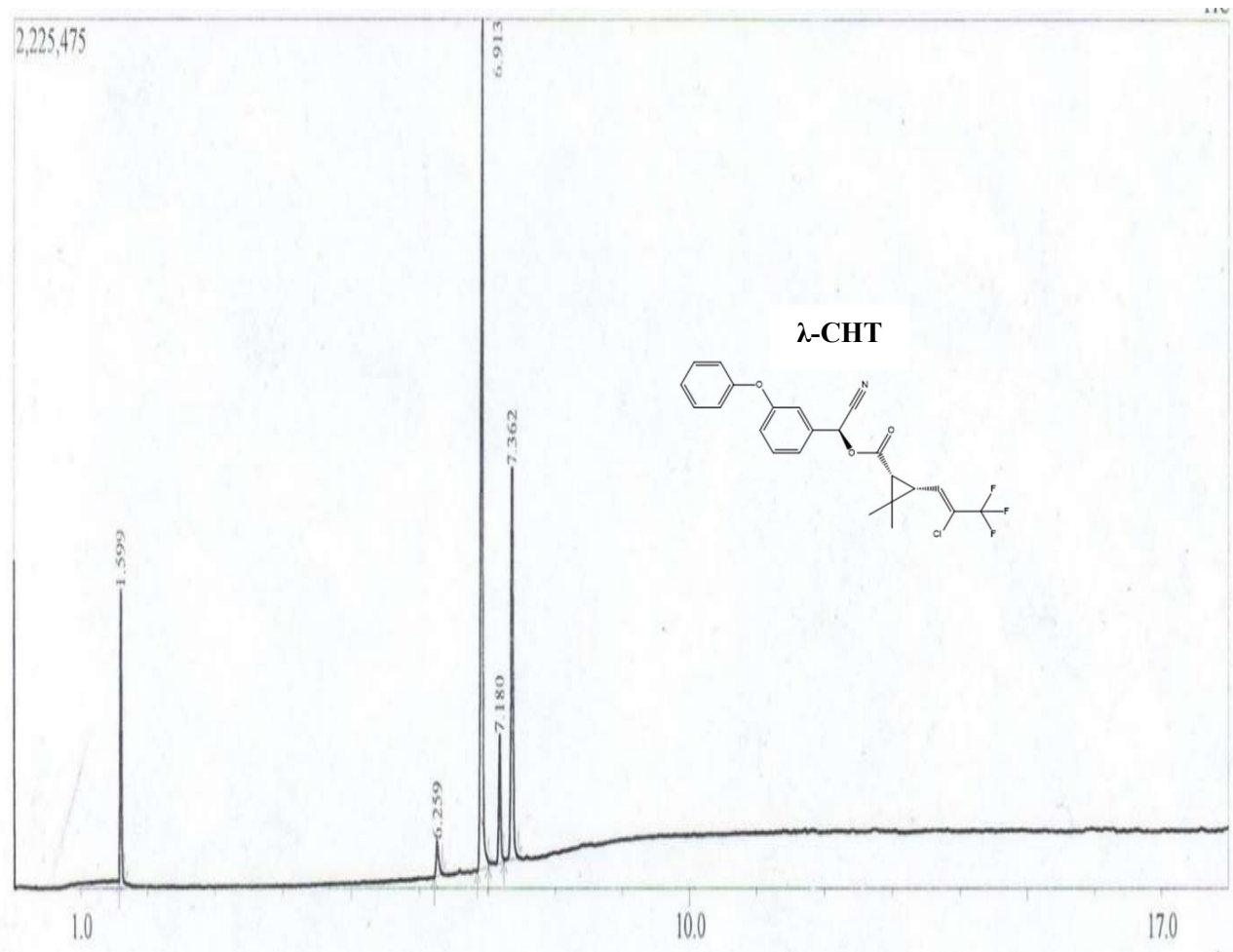

**S2 Fig. GC chromatogram of λ-CH**

Supplement: S2 Fig — (PDF) [file pone.0258864.s002.pdf]
